# Supplementary material for: Patterns of Intron Gain and Loss in Fungi
Source: PLoS Biol. 2004 Nov 30;2(12):e422. doi: 10.1371/journal.pbio.0020422 (PMC532390; doi:10.1371/journal.pbio.0020422)
Supplement: Table S1 — Also available at http://genes.mit.edu/NielsenEtAl/. (4.3 MB ZIP). [file pbio.0020422.st001.zip › NielsenEtAl/html/431.html]

AN3168.1.NCU03395.1.MG07007.1.FG10907.1


```
 CLUSTAL W (1.82) Multiple Sequence Alignments - Introns Inserted


Sequence 1: NCU03395.1	364 aa
Sequence 2: MG07007.1	366 aa
Sequence 3: FG10907.1	364 aa
Sequence 4: AN3168.1	362 aa
Alignment Length: 366 aa
Number Identitical Residues: 267 aa
Alignment Score (without introns) 11516


MG07007.1 	MEGLLFNVNNG2YIEGIVRGYRNGLLTGSNYANMTQCESVDD1LKLQLGPSYGDFLASLP
NCU03395.1	MEGLLFNVNNG2YIEGIVRGYRNSLLTSTNYTNMTQCESIDD1LKLQLGPAYGDFLASLP
FG10907.1 	MEGLYYNVNNG2YVEGIVRGYRNSLLTGAAYNNLTQCETIDD1LKLQLGPSYGDFLASLP
AN3168.1  	MEGLFFNVNSG2YIEGIVRGYRNSLLNGQHYSNLTQCETIDD1VKLQLAPAYGDFLGALP
          	**** :***.* *:*********.**..  * *:****::** :****.*:*****.:**

MG07007.1 	PNPSTSALAAKATDKLVSEFRYVRANAVGSLAQFMDYLTYGYMIDNVALLITGTLHERDT
NCU03395.1	PKPSTSALAAKTTDKLVSEFRYVRANAAGSLAKFMDYLTYGYMIDNVALLITGTLHERDT
FG10907.1 	PNPSTSALAAKTTDKLISEFRYVRAQAVGSLATFMDYVTYGYMIDNVALLITGTLHERDT
AN3168.1  	PNPSTSALAGKMTDKLVAEFRYLLAQATGSTARFLEYLTYGYMIDNVALLITGTLHERDT
          	*:*******.* ****::****: *:*.** * *::*:**********************

MG07007.1 	RELLERCHPLGWFETMPVLCVATNIEELYNSVLIETPLAPYFKNSLSHTDLDELNIEIVR
NCU03395.1	RELLERCHPLGWFETMPVLCVATNIEELYNSVMIETPLAPYFKSSLSLQDLDELNIEIVR
FG10907.1 	RELLDRCHPLGWFETMPVLCVATNIEELYNSVLIETPLAQYFKGSLSHQDLDELNIEIVR
AN3168.1  	RELLERCHPLGWFETLPVLCVATNIEELYNSVLVETPLAGFFKGSLSHQDLDELNIEIVR
          	****:**********:****************::***** :**.***  ***********

MG07007.1 	NTLYKNYLEDFHNFVNTHPDMAGTPTAEVMSEILEFEADRRAINITLNSFGTELTKEDRK
NCU03395.1	NTLYKNYLEDFYHFVNTHPDMAGTPTAEVMSELLEFEADRRAINITLNSFGTELSKADRK
FG10907.1 	NTLYKNYLEDFYNFVNTHPEMAGTPTAEVMSEILEFEADRRAINITLNSFGTDLSKQDRN
AN3168.1  	NTLYKNYLEDFYKFVTTHPDFKGTPTQEVMAEILEFEADRRAINITLNSFGTELSKPERR
          	***********::**.***:: **** ***:*:*******************:*:* :*.

MG07007.1 	KLYPTFGRLYPEGTLMLSRADDLDGVRLAVDGVSDYKSFFDTAGLSGGLSGPGNMGGGQG
NCU03395.1	KLYPNFGQLYPEGTLMLSRADDFEGVRLAVEGVADYKSFFDAAGLGGGPSGPGNMGGG--
FG10907.1 	KLYPSFGKLYPEGTLMLSRAEDPEGVRLAVDGVHDYKSFFDAINVGGGPSGPGNMGGG--
AN3168.1  	KLYPEFGKLYPEGSLMLSRADDIEAVSLAVSISADYKAFFDAVGLTQGGGGLGGMGGS--
          	**** **:*****:******:* :.* ***.   ***:***: .:  * .* *.***.  

MG07007.1 	GSEGKSLEDMFYQKEMEISKSAFTRQFTFAIVYAWVKLREQ~EIRNITWIAECIAQNQKE
NCU03395.1	GTEGKSLEDMFYQKEMEISKMAFTRQFTYAIVYAWVKLREQ0EIRNITWIAECIAQNQKE
FG10907.1 	AGETKTLEDMFYQKEMEISKNAFTRQFTHGIVYAWVKLREQ0EIRNITWIAECIAQNQKE
AN3168.1  	--DGKSLEDLFYQKEMEMSKLVFTRQFTPAIVYAWMRLKEQ0EIRNVTWISECIAQNQKE
          	  : *:***:*******:** .****** .*****::*:** ****:***:*********

MG07007.1 	RIGNYISVF
NCU03395.1	RINNYISVF
FG10907.1 	RIGNYISVF
AN3168.1  	RIGNFISVF
          	**.*:****
```
